# Maxillonasal dysplasia (Binder's syndrome) and its treatment with costal cartilage graft: A follow-up study

**DOI:** 10.4103/0970-0358.44925

**Published:** 2008

**Authors:** Yogesh C. Bhatt, Kinnari A. Vyas, Mangesh S. Tandale, Nikhil S. Panse, Harpreet S. Bakshi, Rajat K. Srivastava

**Affiliations:** Department of Plastic Surgery, SSG Hospital and Medical College, Baroda, India

**Keywords:** Binder's syndrome, costal cartilage grafts, nose correction, premaxillary augmentation

## Abstract

Maxillonasal dysplasia or Binder's syndrome is an uncommon congenital condition characterized by a retruded mid-face with an extremely flat nose. We report here six patients with maxillonasal dysplasia whose noses were corrected with onlay costal cartilage grafts using a combined oral vestibular and external rhinoplasty approach for nasal dorsal augmentation, columellar lengthening, and premaxillary augmentation. The cartilage graft was dipped in a solution of 100 ml 0.9% NaCl and one vial (80mg) gentamicin for 30 min to prevent warping. L struts made for nasal augmentation, columellar lengthening, and premaxillary augmentation were fixed to one another by slots made in the graft. This technique has been used in children, adults, and for secondary cases with promising results. All patients were of class I dental occlusion. The nasal and premaxillary augmentation which was monitored by serial photography was found to be stable over a follow-up period of three years.

## INTRODUCTION

Maxillonasal dysplasia (Binder's syndrome) is a congenital malformation characterized by nasomaxillary hypoplasia due to an underdevelopment of the mid-facial skeleton.[1–3] The etiology of this condition, as suggested by Binder, was a disturbance of the prosencephalic induction center during embryonic life.[2] Birth trauma has also been suggested as a possible causative factor.[4] The essential feature of maxillonasal dysplasia was initially described by Noyes in 1939,[4] although it was Binder who first defined it as a distinct clinical entity in 1962. Binder reported three cases and six characteristic features[5]: (1) arhinoid face; (2) abnormal position of the nasal bones; (3) Intermaxillary hypoplasia with consecutive malocclusion; (4) reduced or absent anterior nasal spine; (5) atrophy of the nasal mucosa, and (6) absence of the frontal sinus (not obligatory). Individuals with Binder's syndrome have a characteristic appearance that is easily recognizable.[6] The mid-face profile is hypoplastic, the nose is flattened, the upper lip is convex with broad philtrum, nostrils are typically crescent or semi lunar-shaped giving a half-moon appearance, columella are short with deep fossa or folds between the upper lip and the nose, resulting in an acute nasolabial angle. The frontonasal angle is almost 180°, resulting in a concave mid-face profile. Since Binder first recognized the syndrome in 1962, well over 250 cases have been reported with equal sex predominance and severity ranging from mild to severe. Cephalometrically, there is reduced sella nasion distance[7] and the length of the maxilla measured from the anterior surface of the maxilla to the posterior nasal spine is reduced. However, it has been suggested that there is a common concurrent induction process for both the prosencephalic area and the vertebrae, accounting for the increase in vertebral anomalies associated with this condition.[8] Maxillonasal dysplasia can also be combined with other malformations. In the most severe cases, the syndrome is associated with true mandibular prognathism requiring combined orthodontic and surgical treatment.[10] There may be pseudomandibular or true mandibular prognathism with a hypoplastic maxilla. The severity of the malocclusion is ultimately connected with the severity of the syndrome. In mild cases, orthodontic treatment may not be necessary because of compensatory effects in dental arches, while in the most severe cases, the maxillary underdevelopment is aggravated by mandibular prognathism and can only be treated by combined orthodontics and surgery. In longitudinal cephalometric studies comparing orthodontically treated children with Binder's syndrome with untreated cases, it was concluded that conventional orthodontic therapy did not produce evidence for a positive influence on craniofacial growth.[10] With increasing age, the maxilla grew forward, but not to the same extent as the mandible. Growth impediment was confined to the area around the absent anterior nasal spine in subjects with moderate forms of the syndrome. Olow-Norderam and Thilander advised postponing definitive orthodontic treatment in individuals with maxillonasal dysplasia until growth has stopped, especially in those with severe malocclusion.[10] It has been suggested that corrective surgery of the mid-face and nose has the potential to jeopardize acceptable occlusal results following early orthodontic correction. Olow-Norderam concluded that the severity of the malocclusion was evident at an early age. Patients who proceeded on to orthognathic surgical correction had more retrognathic maxillae, increased mandibular planes angles, large gonial angles, and markedly negative apical base angles than milder cases with Binder's who were treated orthodontically with success. The possibility of family history was put forward by Ferguson and Thompson.[11] Olow-Norderam reported positive family history in 36% of their patients.[12,13] Gorlin *et al.* suggest that maxillonasal dysplasia is a nonspecific abnormality of the nasomaxillary complex. They believe that familial examples are a result of complex genetic factors, similar to those involved in producing a malocclusion.[14] In the present study, we report our experience on the correction of the nasal and premaxillary areas in six patients with Binder's Syndrome over a follow-up period of three years. We also describe our treatment method using costal cartilage grafts for dorsal nasal augmentation, columellar lengthening, and premaxillary augmentation.

## MATERIALS AND METHODS

Our study is based on six patients with Binder's syndrome treated in our institute in 2004. At the time of initial consultation, the patients' ages ranged from 8 to 25 years with equal sex distribution. Physical examination findings included mid-facial hypoplasia, flattened nose, short columella with an acute nasolabial angle, and retrusion of the anterior nasal spine and fronto nasal angle approaching 180°. All patients had class I dental occlusion with no malalignment of teeth. All patients were evaluated pre- and postoperatively by serial photography; postoperative follow-up ranged from six months to three years with average follow up of one and half years. Our surgical treatment plan consisted of nasal augmentation, columellar lengthening, and premaxillary augmentation using costal cartilage grafts. The grafts were harvested from the right side of the chest through a small submammary incision in females and a lower oblique incision in males. To achieve an anterior projection of the nose and mid-face, usually three cartilaginous strips were implanted through a combined external rhinoplasty and oral vestibular approach. L struts were made for dorsal augmentation and columellar lengthening and a separate one was made for premaxillary augmentation onto the superior alveolar process. The latter was fixed at the maxilla by screws and intergraft fixation was done by slots made in the graft. Another triangular slot was made in the premaxillary strut on the posterior aspect to fit into the anterior nasal spine area for better fixation and to prevent displacement. The dorsal and columella grafts each were carved from the central section of the costal cartilage to prevent warping. Cartilage grafts were dipped in 0.9% NaCl and gentamicin solution to prevent warping, probably because the extracellular matrix of the cartilage is rich in sodium and dipping it in sodium chloride prevents warping.[15]

Figures 1a and 1b show a schematic drawing of cartilage grafting procedure in Maxillonasal dysplasia. Placement of the three cartilaginous splinters: one on the dorsum, the second into the columella, and the third onto the maxilla fixed by screws avoiding dental roots. The arrows point to the fixation at the interlocking of the grafts.

**Figure 1a:**
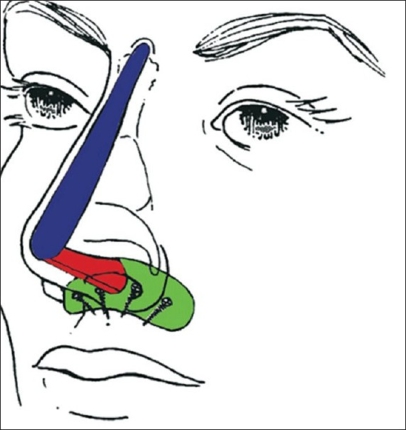
Schematic representation of cartilage graft fixation with screws

**Figure 1b:**
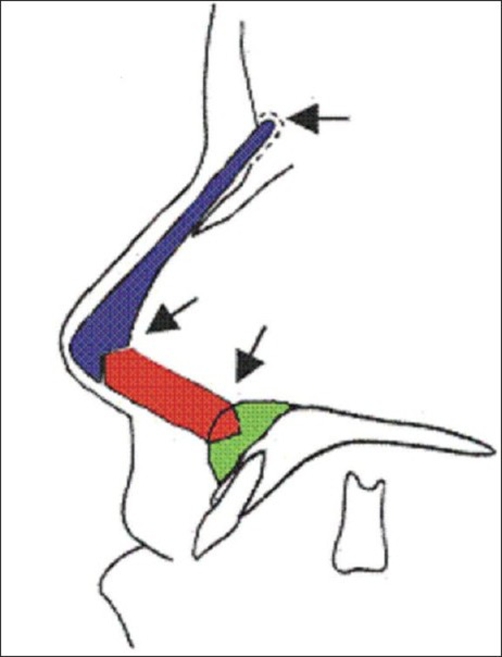
Schematic representation of cartilage graft fixation with screws

Figures 2a, and 2b show: costal cartilage graft carved to the desired shape, three separate struts for dorsal, columellar, and premaxillary portions. Arrow showing slot made in premaxillary segment.

Case 1: Twenty-one year-old male with Binder's maxillonasal dysplasia. Comparison of preoperative state [Figures 3a, 4a, 5a] and postoperative result [Figures 3b, 4b, 5b].

**Figure 2a:**
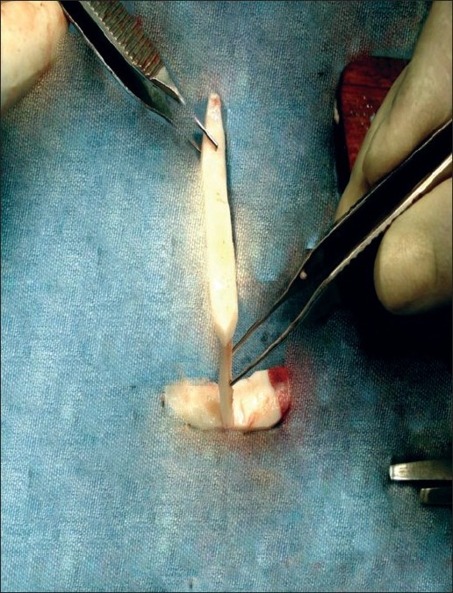
Cartilage graft carved to desired shape

**Figure 2b:**
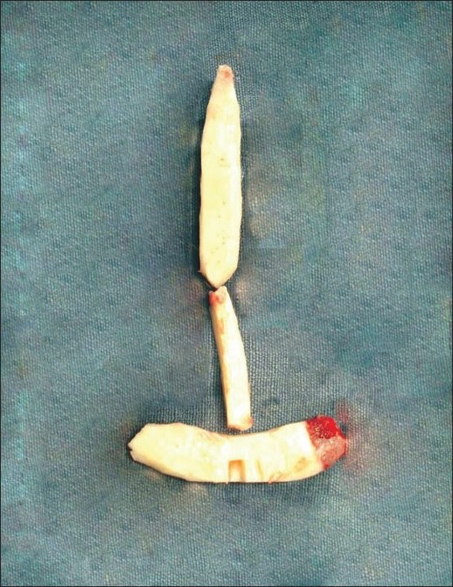
Cartilage graft in desired shape after temporary fixation

**Figure 3a:**
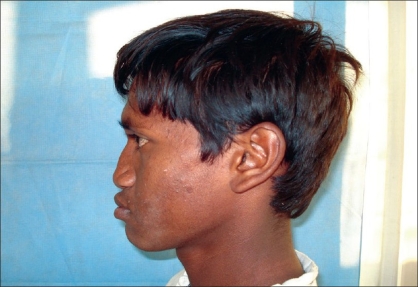
Preoperative photograph in profile view

**Figure 3b:**
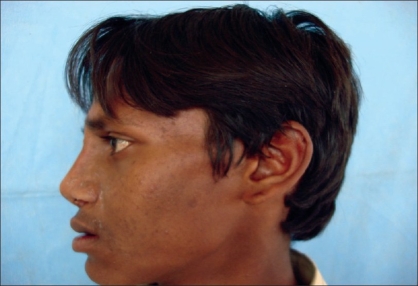
Postoperative photograph in profile view

**Figure 4a:**
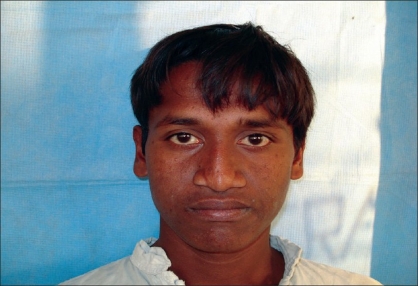
Preoperative photograph in frontal view

**Figure 4b:**
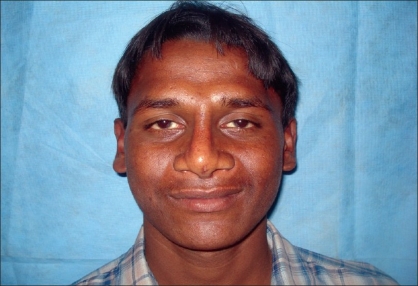
Postoperative photograph in frontal view

**Figure 5a:**
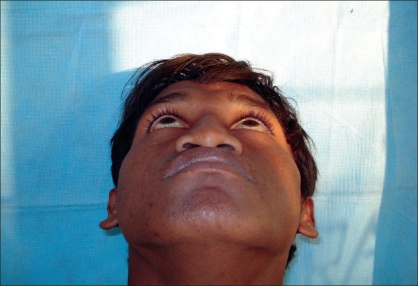
Preoperative photograph in worms eye view

**Figure 5b:**
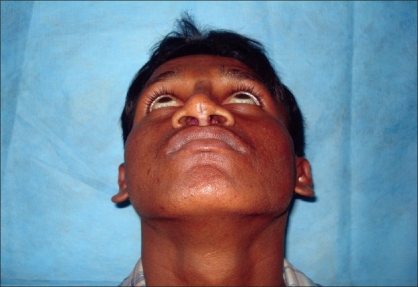
Postoperative photograph in worms eye view

Case 2: Eight year-old male with maxillonasal dysplasia

Figures 6a, 7a showing preoperative status and Figures 6b, 7b showing postoperative results after 18 months, no deviation, resorption, or warping of cartilage graft with excellent patient satisfaction.

Case 3: Twenty-five year-old female with maxillonasal dysplasia, Figures 8a, 9a showing preoperative status and Figures 8b, 9b showing postoperative results.

**Figure 6a:**
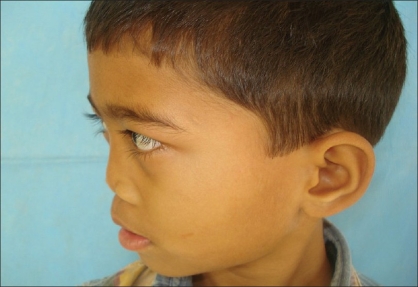
Preoperative photograph in oblique profile view

**Figure 6b:**
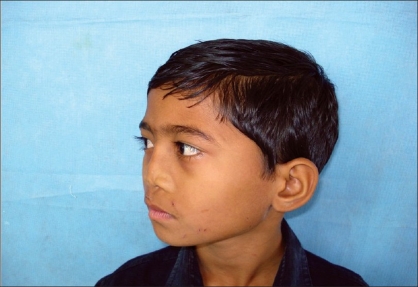
Post operative photograph in oblique profile view

**Figure 7a:**
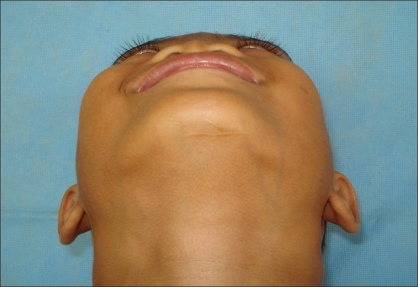
Preoperative photograph in worms eye view

**Figure 7b:**
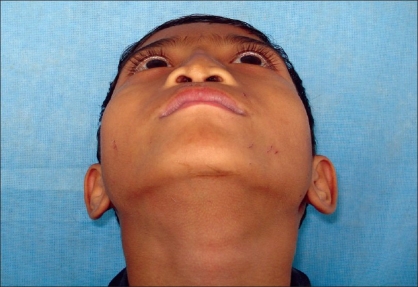
Post operative photograph in worms eye view

**Figure 8a:**
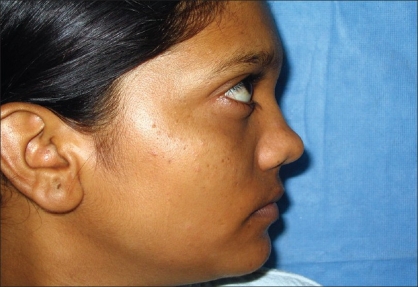
Preoperative photograph in profile view

**Figure 8b:**
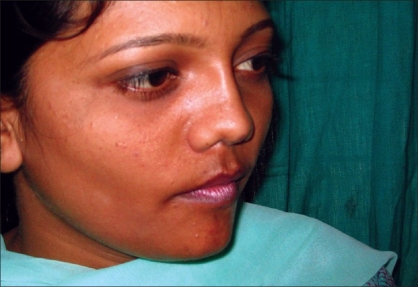
Post operative photograph in profile view

**Figure 9a:**
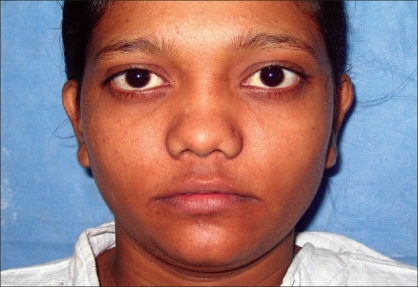
Preoperative photograph in frontal view

**Figure 9b:**
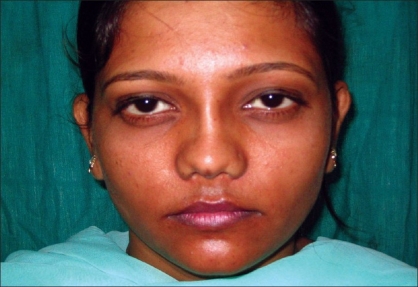
Post operative photograph in frontal view

Case 4: Eighteen year-old female patient operated previously in some another hospital for Binder's syndrome with only nasal augmentation using an iliac crest bone graft showing undercorrection, draft displacement, and deviation. Figures 10a, 11a, 12a showing preoperative status and Figures 10b, 11b, 12b showing postoperative results.

**Figure 10a:**
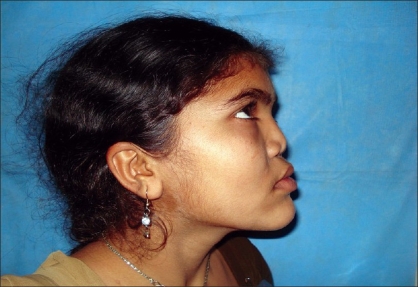
Preoperative photograph in profile view

**Figure 10b:**
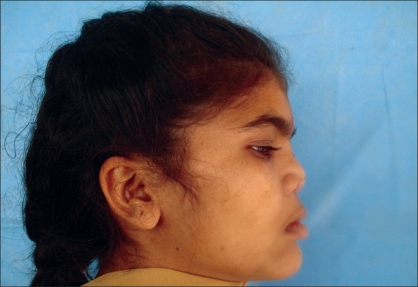
Post operative photograph in profile view

**Figure 11a:**
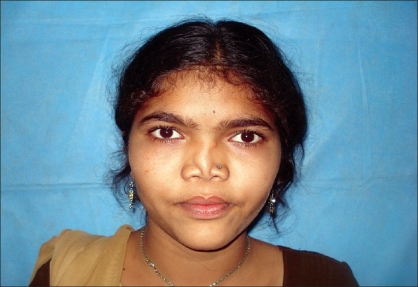
Preoperative photograph in frontal view

**Figure 11b:**
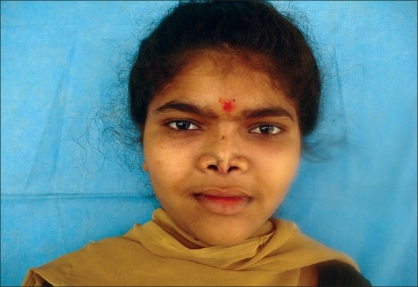
Post operative photograph in frontal view

**Figure 12a:**
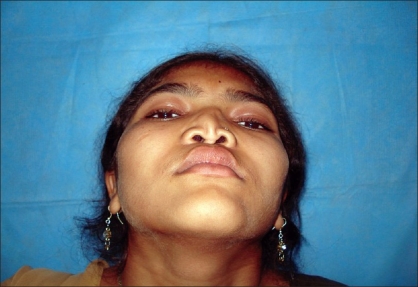
Preoperative photograph in worms eye view

**Figure 12b:**
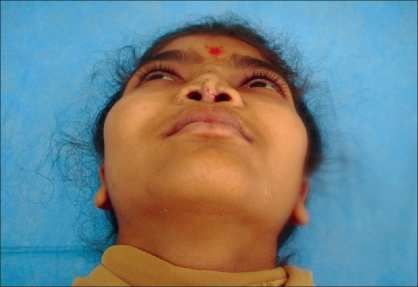
Post operative photograph in worms eye view

## RESULTS

All patients were operated on only once; costal cartilaginous grafts maintained their volume in all areas of the nose regardless of the patient's age. The same was true for the cartilage augmentation to the premaxillary area. In all cases, postoperative clinical follow-up of six months to three years' duration showed the augmentation to be permanent. It must be stressed that by our technique, the mid-face with the nasal base as well as the nose itself were augmented, not just the tip of the nose or only the maxillary bone, respectively. Note also the significant improvement in the length of the columella and the position of the nasal openings. The results showed that the patients' feelings toward their operations were positive. No signs of infection, necrosis, or other complications were observed. The scars were acceptable in all patients at the columella.

## DISCUSSION

In Binder's syndrome, the hypoplasia of the nasal floor and the adjacent part of the maxilla produces the characteristic dish-face anomaly and a flat nose mainly due to a deficient horizontal growth of the maxilla.[16,17,18,19,20,21,22] Surgical correction is demanded as these deformities are evident at a very young age and often lead to severe psychological problems besides the functional restrictions. In planning the treatment strategy, two questions have to be taken into special consideration: (1) what is the appropriate surgery, and (2) which is the optimal age for performing surgery? Bone and cartilage grafts have been traditionally used to treat the maxillonasal hypoplasia. Ragnell described the application of iliac cancellous onlay bone chips to the anterior surface of the maxilla through a median incision at the columellar base.[23] Converse used the oral vestibular approach to insert a shell-like segment of iliac bone.[24] Later, he proposed using an L-shaped bone graft to reconstruct the dorsum and the shortened columella.[25] To raise the nasal contour, Holmström[26] as well as Losken[27] and later Rune,[28] used L-shaped bone grafts taken from the iliac bone and the skull, respectively. They also augmented the premaxillary region with bone chips[26] or a U-shaped bone segment[27] through an oral vestibular approach[26] or a perialar crease incision or one just below the columella.[27] However, the results of bone grafts remain unpredictable. Resorption often occurs especially if the soft tissue cover is very tight and displacement of the bone strut has been described to lead to disappointing long-term results.[28] The patients are very often disturbed by the stiffness of the tip of the nose and the rigidity of the bone implant leads to easier fractures.[16,28] The pain in the bone graft donor site lasts longer and delays ambulation. Costal cartilage grafts, on the other hand, maintain their volume in all areas and produce a more natural feeling to the nose, making it the ideal material for augmentation. To prevent warping of the large grafts required for the dorsum, they must be carved from the central part of the rib and placed into 0.9% sodium chloride solution for about 30 min. Although the balance of the surface tension forces is not maintained, the residual stress of the cartilage splinter comes to the force and even if there is some amount of warping at the end of 30 minutes, it can be taken into account when implanting. Another solution to this fact is the insertion of a thin K wire inside the graft as proposed by Gunter *et al.*[29] So far, we have not seen major problems with cartilage warping in our patients, even after long-term follow-up. Intergraft fixations were done with the help of slots made in the graft which give additional stability and prevent displacement. Ortiz Monasterio *et al.*[30] also described convincing results in augmenting mid-facial deficiencies by using cartilaginous onlay grafts to the pyriform area, such as L-strut grafts for dorsal and columellar areas. Some authors have proposed the use of alloplastic implants but the risk of increased extrusion rates and infection are more as they are not an autogenous material, and it is not cost-effective considering the Indian scenario. The flat nose in Binder's syndrome has also been considered to be a problem of soft tissue deficiency in the columella. Its lengthening has been achieved by the use of a free auricular graft, small flaps from the upper lip, bilateral flaps from the nasal floor, and VY-plasty of the columella.[26] Our concept is to lengthen the columella by VY-plasty if there is a real shortage of skin, but if there is just a retraction into the hypoplastic nasal floor, skin advancement can be achieved by undermining the skin at the lip-columellar junction and with the help of nasal cartilage grafts. If necessary, the cartilaginous septum is rotated forward to additionally support the nasal dorsum. A limitation to the achievement of an optimal result is presented by the constriction of the soft tissue covering the nose and of the lining of the nasal cavities which were not expanded progressively, as it occurs in normal patients. According to Ortiz Monasterio *et al.*,[30] this problem can be prevented if surgical treatment starts early because the corrected facial conditions follow a pattern similar to normal growth. At least equally important is the advantage of improving the self-image of the patients during their growth period when surgery is performed early in life. Therefore, we cannot agree with Tessier *et al.*[31] that the ideal age is 16 years for surgery in Binder's syndrome when growth of the maxilla is completed; one should at least use an onlay graft technique without osteotomies. In our series, all patients had Class one dental occlusion and no malalignment of the teeth, so no orthodontic treatment was required. In cases with severe malocclusion, particularly Type three, maxillary retrognathia should be corrected by a Le Fort one maxillary advancement. However, even if the septum and nasal bones are included in the advanced segment, as in a Le Fort two osteotomy, the flat nose and the depressed alar base remain and with it remain the facial characteristics of Binder's syndrome.[32] This is mainly due to the absent septal support of the nasal dorsum and the relative retrusion of the septum with respect to the nasal base.[25,33] Furthermore, a Le Fort two osteotomy lessens the normal glabellar depression and this may be a limiting factor as a nasal dorsum coming straight off the lower forehead is not ideal aesthetically.[34] These facts point to the major importance of nasal correction in patients with Binder's syndrome. In severe cases of the syndrome, Holmstrüm and Kahnberg[35] recommend a two-stage surgical procedure, firstly maxillary osteotomy followed by the nasal improvement secondarily, both independent of the patient's age. As the degree of malformation in Binder's syndrome varies significantly, surgical correction needs to be individually tailored based on the demonstrated principles.

The onlay grafting technique seems to positively influence facial growth with minor secondary corrections being an option at any time.

## CONCLUSION

Binder's Syndrome: Augmentation of the premaxilla is necessary along with nasal augmentation and columellar lengthening with autogenous costal cartilage grafts for effective treatment. Carving the grafts from a central segment and dipping it in 0.9% of NaCl solution prevents warping and reduces resorption rates. Making slots in the cartilage grafts helps in better fixation of the grafts. Augmentation is enough to give an aesthetically pleasing facial profile in milder cases.
